# Side Effects of Sulfur Dust on the European Grapevine Moth *Lobesia botrana* and the Predatory Mite *Kampimodromus aberrans* in Vineyards

**DOI:** 10.3390/insects11110825

**Published:** 2020-11-23

**Authors:** Federico Tacoli, Elena Cargnus, Pietro Zandigiacomo, Francesco Pavan

**Affiliations:** Department of Agricultural, Food, Environmental and Animal Sciences, University of Udine, Via delle Scienze 206, 3100 Udine, Italy; tacoli@outlook.it (F.T.); elena.cargnus@uniud.it (E.C.); pietro.zandigiacomo@uniud.it (P.Z.)

**Keywords:** Tortricidae, Phytoseiidae, IPM, natural substances, inorganic fungicides, kaolin, mode of action, powdery mildew, sustainable viticulture

## Abstract

**Simple Summary:**

The European grapevine moth *Lobesia botrana* is the most important carpophagous pest in European vineyards. Synthetic insecticides are usually applied for moth control, but their impact on human health and environmental quality requires a reduction in their usage. The exploitation of the side effects of inorganic fungicides of natural origin repeatedly used during the growing season can contribute to achieve this goal. In this study, a possible effect on *L. botrana* infestation of sulfur dust applied to control powdery mildew is tested under laboratory and field conditions. Sulfur dust reduced egg laying by around 80% in both laboratory and field bioassays. In the laboratory, the product showed a slight contact toxicity on eggs and reduced larval settlement. A single field application of sulfur dust during *L. botrana* egg laying reduced larval infestation by approximately 40%. No adverse effects were observed on the predatory phytoseiid mite *Kampimodromus aberrans*.

**Abstract:**

To reduce the impact of synthetic insecticides on human health and the environment, eco-friendly alternatives must be investigated. Knowledge of the side effects on pests and natural enemies of natural products applied to vineyards is very useful. Sulfur dust, which is used in vineyards to control powdery mildew, is investigated in laboratory and field bioassays for its effects on *Lobesia botrana* egg laying, egg hatching, and larval settlement. In field trials, the efficacy of sulfur dust against the two *L. botrana* carpophagous generations is compared with that of *Bacillus thuringiensis* and kaolin, and its side effects on the phytoseiid mite *Kampimodromus aberrans* are evaluated. In the bioassays, sulfur dust reduced female survival by 43%, egg laying by around 80%, egg hatching by 10%, and larval settlement by 55%. In field trials, sulfur dust caused a significant decrease in the number of *L. botrana* larval nests of both generations, even though the efficacy was lower than that of *B. thuringiensis*. No negative effects of sulfur dust on the predatory mite population density was observed. On the basis of these results, in the context of Integrated Pest Management strategies in vineyards, the activity of sulfur dust against *L. botrana* could be exploited by timing its application to the beginning of egg laying.

## 1. Introduction

The European grapevine moth *Lobesia botrana* (Denis & Schiffermüller) (Lepidoptera: Tortricidae) is the most harmful pest in European vineyards and has recently spread to the Americas [1,2]. In north-eastern Italy, the moth usually completes three generations per year, of which the first is anthophagous and the other two are carpophagous [3]. Damage is mostly associated with the second and third generations, which cause yield losses and favor the spread of bunch rots [4,5,6,7].

The control of *L. botrana* is still largely achieved with synthetic insecticides, but concern about human health and environmental quality, the withdrawal of some active ingredients (e.g., organophosphates), and the moth’s resistance to others [8] are driving the push towards the adoption of alternative tools [9].

In the context of Integrated Pest Management (IPM), mating disruption [10,11] and the use of formulations based on *Bacillus thuringiensis* Berliner [12] are the main environmentally safe alternatives to synthetic insecticides against *L. botrana*. However, although their eco-toxicological profile is positive, the adoption of these control tools is not always satisfactory for farmers. Mating disruption does not provide a good control in cases of high population densities, small and irregularly shaped vineyards, and windy regions. Moreover, the cost of mating disruption is higher than synthetic insecticides especially when other grapevine pests such as the carpophagous *Eupoecilia ambiguella* (Hübner) (Lepidoptera: Tortricidae), leafhoppers, and scales must be controlled at the same time. *Bacillus thuringiensis* is rarely used due to its lower efficacy and persistence than those of synthetic insecticides [13]. Additionally, choice of cultivar [14,15,16,17,18] and cultural practices such as controlled nitrogen fertilization [19], bunch-zone leaf removal [20,21], and kaolin applications [22] can help reduce infestations and damage by *L. botrana*.

In European vineyards, phytoseiid mites (Acari) are reported as efficient predators of tetranychid and eriophyoid mites (Acari) and, in absence of prey, can survive by feeding on pollen, fungi, or plant exudates [23,24,25]. In the grape growing area of north-eastern Italy, *Kampimodromus aberrans* (Oudemans) is among the most frequently observed phytoseiid species in vineyards and its persistence is favored by the use of selective pesticides and habitat management strategies [26,27,28,29].

Formulations of wettable or dustable sulfur powders are used widely in viticulture, due to their fungicidal activity against powdery mildew [30], but their effectiveness in controlling phytophagous mites in vineyard and other crops is also known [31,32,33,34,35]. Some preliminary data suggested an efficacy of sulfur dust against *L. botrana* and the leafhopper *Empoasca vitis* (Göthe) (Hemiptera: Cicadellidae) [31]. However, wettable sulfur was not effective against the leafhopper *Erythroneura elegantula* Osborn (Hemiptera: Cicadellidae) [36]. In vineyards, negative side effects of sulfur dust on natural enemies, including phytoseiid mites, have also been observed [36,37,38,39,40,41,42].

On tomato plants covered with sulfur dust, a reduction of *Tuta absoluta* (Meyrick) (Lepidoptera: Gelechiidae) egg laying has been reported [43,44], and a similar effect can be supposed towards *L. botrana* on grapevines. In north-eastern Italy, sulfur dust is traditionally used in vineyards to control powdery mildew between the BBCH 71 and 79 grapevine growth stages (i.e., “fruit set” and “majority of berries touching”, respectively, according to Lorenz et al. [45]) to allow long term coverage inside the bunches. For the early-flowering grapevine cultivars, the start of the *L. botrana* second annual flight occurs a few days before the BBCH 79 growth stage, and therefore we think that a sulfur dust application at this time could help to control the moth.

The aim of this study is to investigate the activity of sulfur dust on *L. botrana*, both in the laboratory and under field conditions, and its side effects on the predatory mite *K. aberrans* in vineyards.

## 2. Materials and Methods

### 2.1. Laboratory Bioassays

#### 2.1.1. *Lobesia botrana* Used in Bioassays

*Lobesia botrana* individuals used in the laboratory and field bioassays were derived from mass rearing of the moth conducted in a climatic chamber at 24 ± 0.5 °C, 70 ± 5% RH, and a photoperiod of 16:8 (L:D). Larvae were fed on an artificial diet [46], and females laid eggs on transparent polyethylene (PE) bags (30 × 15 cm). The moth mass rearing originated from larvae collected in a north-eastern Italian vineyard (Corona di Mariano del Friuli, Gorizia district, 45°55′30″ N, 13°29′44″ E, 40 m a.s.l., cultivar Pinot Gris) located in the same grape-growing area as the vineyards used for the field trials. For egg laying bioassays (Section 2.1.3 and Section 2.2), one-day-old adults from the reared population were placed in PE bags (30 × 15 cm) for mating. Females were collected after 48 h and individually confined for a further 24 h in glass tubes (10 × 3 cm in diameter) where they started to lay eggs. For the bioassay on larval settlement preference (Section 2.1.5), eggs laid on PE bags were used.

#### 2.1.2. Application of Sulfur Dust on Grapevine Berry Surfaces

For the laboratory bioassays (Section 2.1.3, Section 2.1.4 and Section 2.1.5), unripened grapevine berries of cultivar Pinot Gris (BBCH 75) were used. The berries, removed from the bunch with about half a centimeter of petiole, were first dipped in water for one second and then dusted with sulfur (Zolfo ventilato Stella, Pasquale Mormino & Figlio S.R.L, Termini Imerese, Palermo, Italy, 98.5% pure sulfur) to ensure a thin layer of coverage.

#### 2.1.3. Influence of Sulfur Dust on Female Egg Laying

**Figure 1:**
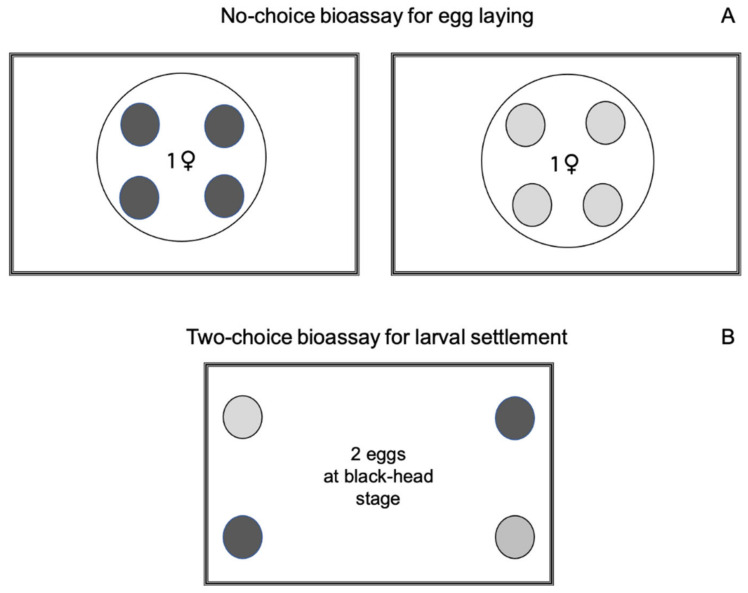
Schemes of laboratory bioassays carried out with sulfur dust on *Lobesia botrana*, as described in Section 2.1.3 (**A**) and Section 2.1.5 (**B**). Rectangle = polystyrene box; large circle = glass Petri dish; dark gray circles = control berries; light gray circles = sulfur-dust-covered berries.

In the laboratory, a no-choice bioassay was conducted under the same climatic conditions described for the moth mass rearing to evaluate whether there was any effect on egg laying by *L. botrana* females when the berries were covered with sulfur dust. The scheme of the bioassay is shown in Figure 1A. The polystyrene box (16 × 9 × 8 cm) was lined with black felt and closed at the top with fine mesh tulle and the box cover on which a little hole was made to guarantee air exchange [47]. Inside each box there was the glass lid of a Petri dish (8 cm diameter) with four equidistant rubber rings glued onto it (1.5 cm external diameter × 1 cm internal diameter × 0.5 cm height). Lid and rubber rings were covered with tulle. The berries were placed on rubber rings, and therefore around 80% of their skin area was still available for egg laying. Felt and tulle were used to avoid oviposition on any surface other than the berries. In the bioassay a box containing four non-covered berries (control berries) was compared with another containing four sulfur-dust-covered berries. The bioassay was replicated 18 times. One fertile female was released into each box, we used individuals that on average had laid a similar amount of eggs inside the glass tubes. The berries were changed daily for five days in both treatments. At each change, it was noted whether females were alive or dead, and the eggs laid by each female on the berry surfaces were counted under a dissecting microscope.

#### 2.1.4. Influence of Sulfur Dust on Egg Hatching

In the laboratory, a bioassay was carried out under the same climatic conditions described for the moth mass rearing to establish the influence of sulfur dust on *L. botrana* egg hatching by comparing the following three treatments: eggs on sulfur-dust-covered berries (i.e., eggs laid after sulfur application), sulfur-dust-covered eggs (i.e., eggs laid within a maximum of 24 h before sulfur application), and eggs laid on untreated berries (control). For the three treatments 155, 188, and 377 eggs were used, respectively. Berries with eggs were placed into cylindrical polystyrene boxes (1.8 × 5 cm in diameter) and after 10 days were checked under a dissecting microscope for egg hatching.

#### 2.1.5. Influence of Sulfur Dust on Larval Settlement Preference

In the laboratory, a two-choice bioassay was conducted under the same climatic conditions described for the moth mass rearing to assess whether any feeding-deterrent effect occurred in *L. botrana* larvae when the berries were covered with sulfur dust (Figure 1B). For this purpose, rectangular polystyrene boxes (9 × 6 × 1.8 cm) were used. To assess larval preference, a strip of PE bag (1 cm^2^) with two eggs at black-head stage was placed in the middle of the box floor with four berries in the corners of each box. Two of these berries were treated with sulfur dust (sulfur-dust-covered berries) and two were untreated (control). To ensure air exchange, a hole (2.5 cm diameter) protected by fine mesh tulle was made in the cover of the box. After 18 h, each box was checked under a dissecting microscope to see which berries newly hatched larvae had settled on. This bioassay was replicated 40 times.

### 2.2. Field Bioassay on Egg-Laying Preference

The influence of sulfur dust on *L. botrana* egg-laying preference was evaluated under field conditions in a two-choice bioassay. Bioassays were carried out from 10 to 20 August (BBCH 89) in a 10-year-old vineyard (Bicinicco, Udine district, 45°55′59″ N, 13°13′60″ E, 35 m a.s.l., cultivar Tocai Friulano) with grapevines grown using the Guyot training system and with distances between and along rows of 2.2 and 0.8 m, respectively. In the vineyard, a standard fungicide program was followed and a single application of acetamiprid (Epik, Sipcam, Milano, Italy) for the control of the leafhopper *Scaphoideus titanus* Ball (Hemiptera: Cicadellidae) was carried out on 20 June, i.e., almost two months before the beginning of the bioassays. Shoots holding two bunches of similar size and not in contact with each other were chosen [21]. Bunches were checked for the absence of *L. botrana* eggs before starting each bioassay. One bunch on each shoot was treated with sulfur dust in order to simulate the distribution of the product at a rate of 30 kg/ha with a field duster machine. During the process, the other bunch (control) was covering with a PE bag for protection. The position of the treated bunch along the shoot was changed in each bioassay to ensure an equal number of proximal and distal bunches in each treatment. Then, each shoot was trimmed and enclosed in a fine mesh tulle cage (25 × 15 cm in diameter) inside which two 1-day-old mated females of *L. botrana* were released. After five days, the cages were removed and bunches harvested. In the laboratory, all berries were checked under a dissecting microscope to count the eggs. The bioassay was replicated 14 times.

### 2.3. Field Trials

#### 2.3.1. Efficacy against *Lobesia botrana*

A total of five trials on the influence of sulfur dust on *L. botrana* larval infestation were carried out in three vineyards (Vineyards A, B and C) in north-eastern Italy. Three and two trials were conducted against the second and third generations of the moth, respectively. Vineyard A (Cormons, Gorizia district, 45°58′32″ N, 13°27′23″ E, 44 m a.s.l., cultivar Pinot Gris) was a 10-year-old organic vineyard with grapevines grown using the Guyot training system with distances between and along rows of 2.4 m and 0.7 m, respectively. Vineyard B (Cormons, Gorizia district, 45°56′32″ N, 13°27′26″ E, 44 m a.s.l., cultivar Tocai Friulano) was a 20-year-old organic vineyard with grapevines grown using the Guyot training system with distances between and along rows of 3.0 m and 1.0 m, respectively. Vineyard C (Bagnaria Arsa, Udine district, 45°52′52″ N, 13°19′17″ E, 17 m a.s.l., cultivar Chardonnay) was a 19-year-old conventional vineyard with grapevines grown using the Sylvoz training system with distances between and along rows of 2.7 m and 1.0 m, respectively. In the three vineyards, standard fungicide programs were followed, and no insecticide was sprayed before or after the product applications.

In Vineyard A (2017 and 2018) and Vineyard C (2020) the efficacy of sulfur dust in the control of *L. botrana* was evaluated against the second generation, whereas in Vineyard B (2017 and 2018) it was evaluated against the third generation. In the trials against the second generation, the treatments were control, sulfur dust (the same product used in the laboratory bioassays), and *B. thuringiensis* (Dipel DF, Sumitomo Chemical Agro Europe S.A.S, Saint Didier au Mont d’Or, Lyon, France, 1% *w*/*v*, Dipel DF/water). In the trials against the third generation, kaolin (Surround WP, Tessenderlo Kerley Inc., Phoenix, AZ, USA, 2% *w*/*v*, Surround WP/water) was also tested alongside the treatments just described. All products were applied using a backpack sprayer (M1200, Cifarelli s.p.a., Voghera, Pavia, Italy). *Bacillus thuringiensis* and kaolin were sprayed at the rate of 1000 L/ha, while sulfur dust was applied at the rate of 30 kg/ha using a kit for powder distribution (OG.346.00, Cifarelli s.p.a., Voghera, Pavia, Italy). Products were applied at defined *L. botrana* phenological stages predicted according to male flights monitored with pheromone traps and average air temperatures. Sulfur dust was applied once at the beginning of egg laying both against the second generation (20 June 2017, 10 June 2018, 19 June 2020) and against the third generation (1 August 2017 and 5 August 2018). Kaolin was applied on two occasions at the beginning of male flight (31 July and 2 August 2017, 3 and 6 August 2018). Against the second generation, *B. thuringiensis* was applied at the beginning of egg hatching and after 7–10 days (22 June and 3 July 2017, 15 and 22 June 2018, 23 June and 2 July 2020), whereas against the third generation, it was only applied at the beginning of egg hatching (1 August 2017, 5 August 2018). When sulfur dust was distributed, the grapevines were at the BBCH 77 growth stage (i.e., “berries beginning to touch”) in the trials against the second generation and at the BBCH 85 growth stage (i.e., “softening of berries”) in the trials against the third generation. In all trials, a randomized block design with four replicates (rows) was adopted. Each row of the Vineyards A, B, and C was divided into three plots of 28 grapevines, four plots of 30 grapevines and three plots of 20 grapevines, respectively.

The level of larval infestation by *L. botrana* was estimated approximately 40 days after the beginning of the pheromone trapping of the males of the second or third annual flights. In each plot of the Vineyards A, B, and C, 100 bunches were sampled from 26, 28, and 18 grapevines, respectively, excluding edge plants. The number of grapevines sampled was different, due to the different number of bunches per grapevine in the three vineyards. The sampling was based on an a priori design [5] to avoid the subjective choice of the sampled bunches. On each bunch, the number of larval nests was counted.

#### 2.3.2. Side Effects on Phytoseiid Mites

In 2018, the toxicity of one application of sulfur dust on phytoseiid mites was studied in a vineyard (Vineyard D) in the same locality as Vineyards A and B. Vineyard D (Cormons, Gorizia district, 45°57′51″ N, 13°26′49″ E, 56 m a.s.l., cultivar Pinot Gris) was a 12-year-old conventional vineyard with grapevines grown using the Guyot training system with distances between and along rows of 2.5 m and 0.8 m.

Preliminary observations revealed the absence of tetranychid mites and the exclusive presence of *K. aberrans* among phytoseiid mites. This phytoseiid species was the only one recorded in Vineyards A and B and the one prevalent in Vineyard C (data not reported). The treatments compared, the products used, the application rate, and the spraying modality were the same as those reported for the trials against the *L. botrana* second generation (Section 2.3.1). To assess phytoseiid population densities, three samplings were carried out: just before applying the products (10 June), just before the second application with *B. thuringiensis* (22 June), and a week after this latter application (29 June). On each sampling date, 10 leaves per replicate (40 leaves per treatment) were collected from the mid parts of the main vine shoots. The leaves were checked under a dissecting microscope to assess predatory mite numbers. At least 100 specimens were slide mounted in Berlese medium and identified under 400× magnification, using the current key [25].

### 2.4. Statistical Analyses

Statistical analyses were performed using IBM SPSS Statistics 20 (IBM Corporation 2011).

For laboratory data, a two-sample *t*-test for the no-choice bioassay (Section 2.1.3), a repeated G-test of goodness-of-fit for the two-choice bioassay (Section 2.1.5.), and a Ryan’s test for proportions comparison (Section 2.1.4) were used. In the no-choice bioassay, the Kaplan–Meier analysis was used to estimate the survival curves on the sulfur dust and control, which were then compared through a log-rank test.

For the two-choice bioassays in the field (Section 2.2), a Wilcoxon matched-pairs signed-ranks test was used.

For trials against *L. botrana* (Section 2.3.1), an ANOVA general linear model was performed considering trial, treatment, replicate, and the interaction trial × treatment as the sources of variation. To satisfy assumptions of a normal distribution, data were square root transformed. Differences among trials and treatments were evaluated using Tukey’s post-hoc tests with Bonferroni adjustment of the *p*-values (α = 0.05).

To compare field data on phytoseiid mites (Section 2.3.2), a mixed ANOVA with Bonferroni adjustment and Tukey’s post-hoc tests were performed after logarithmic transformation of the data, considering treatment as the between-subjects factor and time as the within-subjects factor.

## 3. Results

### 3.1. Laboratory Bioassays on *Lobesia botrana*

#### 3.1.1. Influence of Sulfur Dust on Female Egg Laying

In the no-choice bioassay, *L. botrana* females survived significantly fewer days on sulfur-dust-covered berries (2.50 ± 0.62, mean ± SD) than the control berries (4.39 ± 0.92) (*χ*^2^ = 10.12, df = 1, *p* < 0.001).

**Figure 2:**
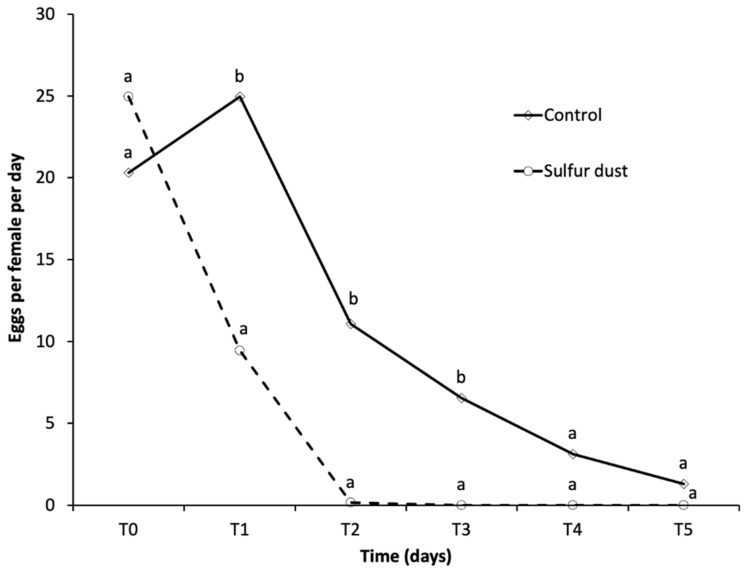
No-choice bioassay. Eggs laid from T1 to T5 by *Lobesia botrana* females on sulfur-dust-covered berries (sulfur dust) and control berries. The eggs at T0 are those laid by the same females on glass tubes the day before the beginning of the bioassay. Different small letters on the same day indicate significant differences according to a two-sample *t* test.

The day before the beginning of the no-choice bioassay (T0), the number (mean ± SD) of *L. botrana* eggs laid inside the glass tubes was not significantly different between the two groups of females used in the sulfur-dust-covered berries (26.90 ± 13.40 eggs/female) and control berries (22.60 ± 18.30 eggs/female) (*t* = 0.81, df = 34, *p* = 0.42) (Figure 2). During the no-choice experiments, females laid significantly fewer eggs in the sulfur-dust-covered berries (11.60 ± 11.48 eggs/female) than the control berries (52.72 ± 36.84 eggs/female) (*t* = 4.52, df = 34, *p* < 0.001). On average, sulfur-dust-covered berries reduced egg laying by 78.0%. The egg-laying pattern from T1 to T5 was different between the sulfur-dust-covered berries and control berries (Figure 2). As a consequence of the different patterns, the number of eggs laid per female was significantly lower in the sulfur-dust-covered berries than control berries from T1 to T3 (T1, *t* = 2.49, df = 38, *p* = 0.017; T2, *t* = 2.80, df = 38, *p* = 0.008; T3, *t* = 2.84, df = 38, *p* = 0.007). In the sulfur-dust-covered berries, no female laid eggs from T3, whereas in the control berries, some females laid eggs up to T5.

#### 3.1.2. Influence of Sulfur Dust on Egg Hatching

The hatching rate of eggs covered by sulfur dust (89.4%) was significantly lower than both the control eggs (99.5%) and untreated eggs laid on sulfur-dust-covered berries (93.5%) (Ryan’s test, *p* < 0.05). On average, the hatching of eggs covered with sulfur dust was reduced by 10%.

#### 3.1.3. Influence of Sulfur Dust on Larval Settlement Preference

A significantly lower number of larvae settled on the sulfur-dust-treated berries (30.8%) than control berries (69.2%) (*G* = 9.87; df = 1, *p* < 0.001). On average, sulfur dust reduced larval preference for settlement by 55%.

### 3.2. Field Bioassay on Egg-Laying Preference of *Lobesia botrana*

In the two-choice bioassay, *L. botrana* females laid eggs on at least one of the two bunches in 11 out of 14 cages. The number of eggs was significantly lower in the sulfur-dust covered bunches (2.36 ± 1.61, mean ± SE eggs per cage) than control ones (11.81 ± 5.29) (W = 66; N = 11, *p* = 0.001). On average, the egg-laying preference on berries in bunches covered with sulfur dust was reduced by 80%.

### 3.3. Field Trials

#### 3.3.1. Efficacy against the Second Generation of *Lobesia botrana*

In the field trials against the second generation, the trial and treatment sources of variation were significant (Table 1). In the trial conducted in 2018, the number of larval nests was significantly higher than in the trials conducted in 2017 and 2020. Sulfur dust and *B. thuringiensis* resulted in significantly fewer larval nests than control, with *B. thuringiensis* significantly more effective than sulfur dust.

#### 3.3.2. Efficacy against the Third Generation of *Lobesia botrana*

In the field trials against the third generation, the trial and treatment sources of variation were significant (Table 2). In the trial conducted in 2018 the number of larval nests was significantly higher than in the trial conducted in 2017. Sulfur dust, *B. thuringiensis*, and kaolin resulted in significantly fewer larval nests than the control, with *B. thuringiensis* significantly more effective than sulfur dust.

#### 3.3.3. Side Effects on *Kampimodromus aberrans*

Confirming the preliminary observations, only *K. aberrans* was recorded. Phytophagous mites were not found.

The population of this predatory mite was significantly influenced by time, resulting in lower density in the first sampling, but not by treatment (Table 3). The interaction time × treatment was not significant.

## 4. Discussion

Under both laboratory and field conditions, sulfur dust reduced the egg laying of *L. botrana* on grape berries by 80% in accordance with the results reported for *T. absoluta* on tomato plants [43,44]. In the field two-choice bioassay, *L. botrana* females laid fewer eggs on sulfur dust bunches than on untreated bunches, suggesting that sulfur dust acts as an oviposition deterrent. In accordance with this hypothesis, in the laboratory no-choice bioassay, females on the sulfur-dust berries laid fewer eggs than females on the control berries, proving that under the conditions of the bioassay, *L. botrana* was not able to overcome the sulfur deterrence. For analogy under field conditions, being all the bunches dusted with sulfur powder, we can expect *L. botrana* females to lay fewer eggs because they cannot find alternative oviposition sites on grapevines. The reduction in egg laying by females exposed to sulfur dust in the laboratory could also be due to their shorter survival, but this effect cannot be considered present and consistent even under field conditions. In fact, the increase in mortality of females under laboratory conditions could be due sulfur sublimating inside the boxes. About the causes of the deterrent effect, it can be hypothesized that sulfur dust acts as a physical barrier to oviposition or as a repellent. The possibility that a dust could hinder *L. botrana* egg laying by changing the berry surface from smooth to dusty and irregular was previously supposed for kaolin [22]. The repellence of sulfur dust, and perhaps its toxicity, for females was supposed for *T. absoluta* [43].

The coverage of *L. botrana* eggs with sulfur dust under laboratory conditions reduced hatching slightly, but the impact of this effect under field conditions should be negligible considering (i) the timing of sulfur dust application at the beginning of egg laying, i.e., when few eggs are present, and (ii) the low mortality of sulfur-covered-eggs (around 10% in the laboratory). It is only by timing the application of sulfur dust to the peak of egg laying that a significant percentage of eggs could be covered by the product.

In the laboratory, sulfur dust reduced the preference of *L. botrana* larval settlement in line with the results obtained for *T. absoluta* [43], but in this earlier study the effect was stronger (around 90% compared to 55%). The deterrent effect of sulfur dust on the larvae, markedly observed in the laboratory, could also occur in the field and hinder the penetration of the berries by the newly hatched larvae, which, when remaining longer on the skin of the grapes, could be more exposed to the abiotic and biotic factors of mortality [21]. The prolonged wandering time of the newly hatched larvae could also increase the efficacy of insecticides active exclusively or predominantly before the larvae penetrate the berries (e.g., *B. thuringiensis,* indoxacarb), lengthening the period in which they can be in contact with or feed on the skin of the berries.

In our field trials, the effectiveness (i.e., reduction of larval infestation) of a single application of sulfur-dust against *L. botrana* was approximately 40%. With all the limitations of a rough comparison, the efficacy of the same product used against *T. absoluta* was negligible with two applications [43] and reached 90% with seven treatments [44].

To maximize the positive side effects of a natural substance against grapevine pests, they must be timed to the most susceptible phenological stages of the insects. For *L. botrana* control, kaolin used in vineyards to reduce berry sunburn damage should be applied at the beginning of egg laying of the moth [22], but the same substance for controlling the leafhopper *E. vitis* must be applied in presence of the early nymphal instars [48]. In the case of sulfur dust, because the most important mode of action against *L. botrana* is a reduction in egg laying, the timing that gives the best side effect on *L. botrana* populations is an application at the beginning of egg laying. Preliminary field data had suggested an efficacy of sulfur dust against *L. botrana*, applying it a long time before egg laying [31]. On the basis of the present study, the optimal time for applying sulfur dust against powdery mildew in vineyards, which is traditionally just before the occurrence of the BBCH 79 growth stage (i.e., “majority of berries touching each other”), can be adjusted to coincide with the second-generation egg laying by *L. botrana*. As observed in our *L. botrana* mass rearing (data not reported), this oviposition begins about five days after the start of *L. botrana* male flight, as established by checking pheromone traps daily.

Sulfur dust had a lower efficacy than *B. thuringiensis* not only against the second generation of *L. botrana*, when the latter was applied twice, but also against the third generation, when the bioinsecticide was applied only once. Therefore, sulfur dust cannot be considered an alternative to *B. thuringiensis* against *L. botrana*, but its side effect can integrate other control measures such as mating disruption and *B. thuringiensis* itself. In particular, the application of sulfur dust in combination with *B. thuringiensis* could have even a synergistic effect in *L. botrana* control if an increasing in wandering time of newly-hatched larvae occurs. Sulfur dust showed an efficacy against *L. botrana* similar to that of kaolin, whose activity against the moth, already observed previously [22], was confirmed.

In the literature, negative effects of sulfur dust on non-target arthropods, particularly phytoseiid mites, have been reported [36,37,38,39,40,41,42]. In this study, a single field application of sulfur dust at the rate of 30 kg/ha did not show any detrimental effect on *K. aberrans*. No or low toxicity to predatory mites was reported also in other studies [49,50,51,52,53]. Furthermore, kaolin can negatively affect phytoseiid mites, but this effect was not persistent [54].

## 5. Conclusions

This study highlights that sulfur dust used against powdery mildew can have a valuable side effect on *L. botrana* control. Moreover, the field trials confirmed the efficacy of kaolin against the moth.

In the context of IPM strategies, the possibility of exploiting positive side effects of natural substances should be encouraged, but the maximum advantage is obtained by timing them to the most susceptible phenological stages of the pests.

In this study, the positive effects of a single application of sulfur dust in *L. botrana* and powdery mildew control were obtained without negative effects on the *K. aberrans* population and consequently no risk of phytophagous mites’ outbreaks can be expected.

From a general point of view, sulfur and other natural substances could also negatively affect some natural enemies, however, in the context of IPM strategies, every cultural practice must be evaluated by balancing positive and negative effects.

## Figures and Tables

**Table 1:** *Lobesia botrana* second generation. Larval nests per 100 bunches recorded in the three field trials conducted in 2017, 2018, and 2020, respectively.

Source of Variation	Levels	Mean ± SE	F	Degrees of Freedom	*p*
Trial	2017	8.83 ± 2.64 a	27.59	2, 24	<0.0001
	2018	30.50 ± 7.56 b			
	2020	5.75 ± 1.48 a			
Treatment	Control	24.67 ± 6.38 c	16.39	2, 24	<0.0001
	Sulfur dust	15.25 ± 6.75 b			
	*Bacillus thuringiensis*	7.17 ± 2.01 a			
Replicate			1.61	3, 24	0.21
Trial × treatment			0.99	4, 24	0.43

F and *p* are ANOVA values. Different small letters among the larval nests recorded in the three years or treatments indicate significant differences according to Tukey’s test (α = 0.01).

**Table 2:** *Lobesia botrana* third generation. Larval nests per 100 bunches recorded in the two field trials conducted in 2017 and 2018, respectively.

Source of Variation	Levels	Mean ± SE	F	Degrees of Freedom	*p*
Trial	2017	7.00 ± 1.41	242.52	1, 21	<0.0001
	2018	35.50 ± 3.73			
Treatment	Control	35.25 ± 8.17 c	22.76	3, 21	<0.0001
	Sulfur dust	21.75 ± 6.45 b			
	Kaolin	15.25 ± 4.16 ab			
	*Bacillus thuringiensis*	12.75 ± 3.65 a			
Replicate			0.69	3, 21	0.57
Trial × treatment			1.90	3, 21	0.16

F and *p* are ANOVA values. Different small letters among the larval nests recorded in the four treatments indicate significant differences according to Tukey’s test (α = 0.01).

**Table 3:** Side-effects of sulfur dust on the phytoseiid mite *Kampimodromus aberrans.* Motile forms per leaf recorded on three dates in 2018. On 10 June, sampling was undertaken just before the application of sulfur dust and *B. thuringiensis* and on 22 June, just before the second application of the bio-insecticide.

Source of Variation	Levels	Mean ± SE	F	Degrees of Freedom	*p*
Time	10 June	9.46 ± 1.42 a	19.89	2, 18	<0.0001
	22 June	14.66 ± 1.82 b			
	29 June	14.14 ± 1.96 b			
Treatment	Control	13.09 ± 1.80	0.18	2, 9	0.84
	Sulfur dust	12.82 ± 1.80			
	*Bacillus thuringiensis*	12.35 ± 2.00			
Time × Treatment			1.37	4, 18	0.282

F and *p* are ANOVA values. Different small letters among the phytoseiid mites recorded in the three dates indicate significant differences according to Tukey’s test (α = 0.01).

## References

[1] Gilligan T.M., Epstein M.E., Passoa S.C., Powell J.A., Sage O.C., Brown J.W. (2011). Discovery of *Lobesia botrana* ([Denis & Schiffermuller]) in California: An invasive species new to North America (Lepidoptera: Tortricidae). Proc. Entomol. Soc. Wash..

[2] Ioriatti C., Lucchi A., Varela L.G., Bostanian N.J., Vincent C., Isaac R. (2012). Grape berry moths in Western European vineyards and their recent movement into the New World. Arthropod Management in Vineyards: Pest, Approaches, and Future Directions.

[3] Pavan F., Floreani C., Barro P., Zandigiacomo P., Dalla Montà L. (2013). Occurrence of two different development patterns in *Lobesia botrana* (Lepidoptera: Tortricidae) larvae during the second generation. Agric. For. Entomol..

[4] Fermaud M., Giboulot A. (1992). Influence of *Lobesia botrana* larvae on field severity of Botrytis rot of grape berries. Plant Dis..

[5] Pavan F., Girolami V., Sacilotto G. (1998). Second generation of grape berry moths, *Lobesia botrana* (Den. & Schiff.) (Lep., Tortricidae) and *Eupoecilia ambiguella* (Hb.) (Lep., Cochylidae): Spatial and frequency distributions of larvae, weight loss and economic injury level. J. Appl. Entomol..

[6] Moschos T. (2006). Yield loss quantification and economic injury level estimation for the carpophagous generations of the European grapevine moth *Lobesia botrana* Den. et Schiff. (Lepidoptera: Tortricidae). Int. J. Pest. Manag..

[7] Pavan F., Bigot G., Cargnus E., Zandigiacomo P. (2014). Influence of the carpophagous generations of the European grapevine moth *Lobesia botrana* on grape bunch rots. Phytoparasitica.

[8] Civolani S., Boselli M., Butturini A., Chicca M., Fano E.A., Cassinelli S. (2014). Assessment of insecticide resistance of *Lobesia botrana* (Lepidoptera: Tortricidae) in Emilia-Romagna region. J. Econ. Entomol..

[9] Lucchi A., Benelli G. (2018). Towards pesticide-free farming? Sharing needs and knowledge promotes Integrated Pest Management. Environ. Sci. Pollut. Res..

[10] Ioriatti C., Lucchi A. (2016). Semiochemical strategies for tortricid moth control in apple orchards and vineyards in Italy. J. Chem. Ecol..

[11] Lucchi A., Ladurner E., Iodice A., Savino F., Ricciardi R., Cosci F., Conte G., Benelli G. (2018). Eco-friendly pheromone dispensers—A green route to manage the European grapevine moth?. Environ. Sci. Pollut. Res..

[12] Ifoulis A.A., Savopoulou-Soultani M. (2004). Biological control of *Lobesia botrana* (Lepidoptera: Tortricidae) larvae by using different formulations of *Bacillus thuringiensis* in 11 vine cultivars under filed conditions. J. Econ. Entomol..

[13] Boselli M., Scannavini M., Melandri M. (2000). Confronto fra strategie di difesa contro la tignoletta della vite. Inf. Agrar..

[14] Fermaud M. (1998). Cultivar susceptibility of grape berry clusters to larvae of *Lobesia botrana* (Lepidoptera: Tortricidae). J. Econ. Entomol..

[15] Moreau J., Benrey B., Thiéry D. (2006). Grape variety affects larval performance and also female reproductive performance of the European grapevine moth *Lobesia botrana* (Lepidoptera: Tortricidae). Bull. Entomol. Res..

[16] Sharon R., Zahavi T., Soroker V., Harari A.R. (2009). The effect of grapevine cultivars on *Lobesia botrana* (Lepidoptera: Tortricidae) population levels. J. Pest. Sci..

[17] Pavan F., Stefanelli G., Villani A., Cargnus E. (2018). Influence of grapevine cultivar on the second generations of *Lobesia botrana* and *Eupoecilia ambiguella*. Insects.

[18] Rid M., Markheiser A., Hoffmann C., Gross J. (2018). Waxy bloom on grape berry surface is one important factor for oviposition of European grapevine moths. J. Pest. Sci..

[19] Vartholomaiou A.N., Navrozidis E.I., Payne C.C., Salpiggidis G.A. (2008). Agronomic techniques to control *Lobesia botrana*. Phytoparasitica.

[20] Pavan F., Cargnus E., Kiaeianmoosavi S., Bigot G., Tacoli F., Zandigiacomo P. (2016). Bunch-zone leaf removal of grapevines to prevent damage by *Lobesia botrana* and grey mould. Bull. Insectol..

[21] Kiaeian Moosavi F., Cargnus E., Pavan F., Zandigiacomo P. (2018). Effects of grapevine bunch exposure to sunlight on berry surface temperature and *Lobesia botrana* (Lepidoptera: Tortricidae) egg laying, hatching and larval settlement. Agric. For. Entomol..

[22] Tacoli F., Cargnus E., Kiaeian Moosavi F., Zandigiacomo P., Pavan F. (2019). Efficacy and mode of action of kaolin and its interaction with bunch-zone leaf removal against *Lobesia botrana* on grapevines. J. Pest. Sci..

[23] Duso C., Pozzebon A., Kreiter S., Tixier M.-S., Candolfi M.P., Bostanian N.J., Vincent C., Isaacs R. (2012). Management of phytophagous mites in European vineyards. Arthropod Management in Vineyards: Pests, Approaches, and Future Directions.

[24] McMurtry J.A., De Moraes G.J., Sourasso N.F. (2013). Revision of the lifestyles of phytoseiid mites (Acari: Phytoseiidae) and implications for biological control strategies. Syst. Appl. Acarol..

[25] Tixier M.S., Baldassar A., Duso C., Kreiter S. (2013). Phytoseiidae in European grape (*Vitis vinifera* L.): Bio-ecological aspects and keys to species (Acari: Mesostigmata). Zootaxa.

[26] Pozzebon A., Borgo M., Duso C. (2010). The effects of fungicides on non-target mites can be mediated by plant pathogens. Chemosphere.

[27] Pozzebon A., Tirello P., Moret R., Pederiva M., Duso C. (2015). A fundamental step in IPM on grapevine: Evaluating the side effects of pesticides on predatory mites. Insects.

[28] Tirello P., Pozzebon A., Duso C. (2013). The effect of insecticides on the non-target predatory mite *Kampimodromus aberrans*: Laboratory studies. Chemosphere.

[29] Tixier M.-S. (2018). Predatory mites (Acari: Phytoseiidae) in agro-ecosystems and conservation biological control: A review and explorative approach for forecasting plant-predatory mite interactions and mite dispersal. Front. Ecol. Evol..

[30] Bencivelli A., Rapparini G. (1975). The activity of chemicals of known and of recent formulation against grapevine powdery mildew. Inf. Fitopatol..

[31] Touzeau J. Les actions secondaires du soufre sur les maladies et ravageurs de la vigne. Proceedings of the International Symposium Elemental Sulphur in Agriculture.

[32] Michelatti G., Pinoggi G., Schreiber G., Mozzone G.C. (1994). Esperienze di lotta razionale contro l’eriofide del nocciolo (*Phytocoptella avellanae* Nal.) condotte nell’arco di un quinquennio in Piemonte. Acta Hortic..

[33] Goebel O., Vergnet C., Heller J.J. (2001). Lutte contre l’érinose et l’acariose de la vigne: Intérêt des traitements de début de saison au soufre. Phytoma.

[34] Ahmed M., Hoque A.K.M.R., Mamun M.S.A. (2011). Efficacy of different sulphur formulations against Red Spider Mite (*Oligonychus coffeae* Nietner) of tea in Bangladesh. Int. J. Sust. Agric. Technol..

[35] Haddadi A., Mirfakhraei S., Aramideh S. (2019). Effects of abamectin, volk oil, detergent and sulfur in control of grape erineum mite, *Colomerus vitis* Pagenstecher (Acari: Eriophyidae) in vineyards of West-Azerbaijan Province, Iran. Ann. Biol..

[36] Jepsen S.J., Rosenheim J.A., Bench M.E. (2007). The effect of sulfur on biological control of the grape leafhopper, *Erythroneura elegantula*, by the egg parasitoid *Anagrus erythroneurae*. BioControl.

[37] Cerutti F., Roux O., Delucchi V. (1989). L’énigme de la nuisibilité de la cicadelle de la vigne au Tessin. Mitt. Schweiz. Entomol. Ges..

[38] Schwartz A. (1993). Occurrence of natural enemies of phytophagous mites on grapevine leaves following application of fungicides for disease control. S. Afr. J. Enol. Vitic..

[39] Hanna R., Zalom F.G., Wilson L.T., Leavitt G.M. (1997). Sulfur can suppress mite predators in vineyards. Calif. Agric..

[40] Hassan S.A., Bigler F., Bogenschütz H., Boller E., Brun J., Calis J.N.M., Coremans-Pelseneer J., Duso C., Grove A., Heimbach U. (1994). Results of the sixth joint pesticide testing programme of the IOBC/WPRS-Working Group «Pesticides and Beneficial Organisms». Entomophaga.

[41] Bernard M.B., Cole P., Kobelt A., Horne P.A., Altmann J., Wratten S.D., Yen A.L. (2010). Reducing the impact of pesticides on biological control in Australian vineyards: Pesticide mortality and fecundity effects on an indicator species, the predatory mite *Euseius victoriensis* (Acari: Phytoseiidae). J. Econ. Entomol..

[42] Gadino A.N., Walton V.M., Dreves A.J. (2011). Impact of vineyard pesticides on a beneficial arthropod, *Typhlodromus pyri* (Acari: Phytoseiidae), in laboratory bioassays. J. Econ. Entomol..

[43] Zappalà L., Siscaro G., Biondi A., Mollá O., González-Cabera J., Urbaneja A. (2012). Efficacy of sulphur on *Tuta absoluta* and its side effects on the predator *Nesidiocoris tenuis*. J. Appl. Entomol..

[44] Zakher A.G., Abdel-Aziz M.A., Afsah A.F.E., Farha H.F. (2016). Response of tomato plants to some agricultural and chemical treatments on fruit yield and its quality relation to *Tuta absoluta* (Meyrick) and *Bemisia tabaci* (Genn.) infestation. Arab Univ. J. Agric. Sci..

[45] Lorenz D.H., Eichhorn K.W., Bleiholder H., Klose R., Meier U., Weber E. (1995). Growth stages of the grapevine: Phenological growth stages of the grapevine (*Vitis vinifera* L. ssp vinifera). Codes and descriptions according to the extended BBCH scale. Aust. J. Grape Wine Res..

[46] Rapagnani M.R., Caffarelli V., Barlattani M., Minelli F. (1990). Descrizione di un allevamento, in laboratorio, della tignoletta dell’uva *Lobesia botrana* Den. e Schiff. (Lepidoptera—Tortricidae) su un nuovo alimento semi-sintetico. Boll. Ist. Ent. G. Grandi Univ. Bologna.

[47] Maher N., Thiéry D. (2004). A bioassay to evaluate the activity of chemical stimuli from grape berries on the oviposition of *Lobesia botrana* (Lepidoptera: Tortricidae). Bull. Entomol. Res..

[48] Tacoli F., Pavan F., Cargnus E., Tilatti E., Pozzebon A., Zandigiacomo P. (2017). Efficacy and mode of action of kaolin in the control of *Empoasca vitis* and *Zygina rhamni* (Hemiptera: Cicadellidae) in vineyards. J. Econ. Entomol..

[49] Hoy M.A., Standow K.A. (1981). Resistance to sulfur in a vineyard spider mite predator. Calif. Agric..

[50] Papaioannou-Souliotis P., Markoyiannaki-Printziou D., Tsagkarakou A., Rumbos I., Adamopoulos I. (1998). Effects of different fungicides and insecticides on populations of *Phytoseius finitimus* (Ribaga) in vineyard in four regions of Greece. Redia.

[51] Rumbos I.C., Papaioannou-Souliotis P., Markoyiannaki-Printziou D., Adamopoulos I.C. (2000). Promotion of integrated pest control system in viticulture in Greece with respect to predatory mites. IOBC WPRS Bull..

[52] Costello M.J. (2007). Impact of sulfur on density of *Tetranychus pacificus* (Acari: Tetranychidae) and *Galendromus occidentalis* (Acari: Phytoseiidae) in a central California vineyard. Exp. Appl. Acarol..

[53] Gázquez J.C., López J.C., Baeza E.J., Pérez-Parra J.J., Pérez C., Meca D.E., Navarro S. (2011). Influence of the sulphur application method on pests, diseases and natural enemies in a greenhouse pepper crop. Acta Hortic..

[54] Tacoli F., Cargnus E., Pozzebon A., Duso C., Tirello P., Pavan F. (2019). Side effects of kaolin and bunch-zone leaf removal on predatory mite population (Acari: Phytoseiidae) occurring in vineyards. J. Econ. Entomol..

